# Retraction Note: Synthesis of silver nanoparticles using *Plantago lanceolata* extract and assessing their antibacterial and antioxidant activities

**DOI:** 10.1038/s41598-025-87591-7

**Published:** 2025-01-28

**Authors:** Muhammad Zahir Shah, Zheng-Hui Guan, Ala Ud Din, Amjad Ali, Ata Ur Rehman, Kashif Jan, Shah Faisal, Shah Saud, Muhammad Adnan, Fazli Wahid, Saud Alamri, Manzer H. Siddiqui, Shamsher Ali, Wajid Nasim, Hafiz Mohkum Hammad, Shah Fahad

**Affiliations:** 1 Key Laboratory of Synthetic and Natural Functional Molecule Chemistry of Ministry of Education, Department of Chemistry and Materials Science, Northwest University, Xi’an, 710127 China; 2 Department of Chemistry, University of Buner, Buner, 17290 Pakistan; 3 School of Environmental and Municipal Engineering, Xi’an University of Architecture and Technology, Xi’an, 710051 China; 4 Department of Physics, Riphah International University, Islamabad, 46000 Pakistan; 5 Faculty of Tropical AgriSciences, Czech University of Life Sciences Prague, Prague, Czech Republic; 6 Department of Horticulture, Northeast Agricultural University, Harbin, China; 7 Department of Agriculture, University of Swabi, Swabi, Pakistan; 8 Department of Botany and Microbiology, College of Science, King Saud University, 11451 Riyadh, Saudi Arabia; 9 Department of Soil and Environmental Sciences, Amir Muhammad Khan Campus Mardan, The University of Agriculture, Peshawar, Khyber Pakhtunkhwa Pakistan; 10 Department of Agronomy, University College of Agriculture and Environmental Sciences, The Islamia University of Bahawalpur (IUB), Bahawalpur, Pakistan; 11 Department of Agronomy, Muhammad Nawaz Sharif University of Agriculture, Multan, 66000 Pakistan; 12 Hainan Key Laboratory for Sustainable Utilization of Tropical Bioresource, College of Tropical Crops, Hainan University, Haikou, 570228 China; 13 Department of Agronomy, The University of Haripur, Haripur, 22620 Khyber Pakhtunkhwa Pakistan

Retraction of: *Scientific Reports* 10.1038/s41598-021-00296-5, published online 21 October 2021

The Editors have retracted this Article. Concerns were raised after publication about irregularities in the data in Figures 2 and 5. An investigation by the Publisher determined that the intervals between data points in the spectra in Figure 2 appear to be similar in a way that does not reflect data collected for different samples. The explanation provided by the Authors about these issues and about Figure 5 was not satisfactory. The Editors therefore no longer have confidence in the reliability of the findings reported in this Article.

The Publisher has not been able to obtain current email addresses for authors Muhammad Adnan and Shah Faisal. All other Authors did not respond to correspondence from the publisher regarding this retraction.

